# Exposure to sub-inhibitory concentrations of cefotaxime enhances the systemic colonization of *Salmonella* Typhimurium in BALB/c mice

**DOI:** 10.1098/rsob.150070

**Published:** 2015-10-14

**Authors:** Roberto C. Molina-Quiroz, Cecilia A. Silva, Cristian F. Molina, Lorenzo E. Leiva, Sebastián Reyes-Cerpa, Inés Contreras, Carlos A. Santiviago

**Affiliations:** 1 Laboratorio de Microbiología, Departamento de Bioquímica y Biología Molecular, Facultad de Ciencias Químicas y Farmacéuticas, Universidad de Chile, Santiago, Chile; 2 Centro de InmunoBioTecnología, Programa Disciplinario de Inmunología, Instituto de Ciencias Biomédicas, Universidad de Chile, Santiago, Chile; 3 Center for Adaptation Genetics and Drugs Resistance, Molecular Biology and Microbiology Faculty, Tufts University, Boston, MA, USA; 4 AUSTRAL-Omics, Universidad Austral de Chile, Valdivia, Chile; 5 Laboratorio de Virología, Centro de Biotecnología Acuícola (CBA), Facultad de Química y Biología, Universidad de Santiago de Chile, Santiago, Chile

**Keywords:** antibiotics, *Salmonella*, virulence, anaerobic metabolism

## Abstract

It has been proposed that sub-inhibitory concentrations of antibiotics play a role in virulence modulation. In this study, we evaluated the ability of *Salmonella enterica* serovar Typhimurium (hereafter *S*. Typhimurium) to colonize systemically BALB/c mice after exposure to a sub-inhibitory concentration of cefotaxime (CTX). *In vivo* competition assays showed a fivefold increase in systemic colonization of CTX-exposed bacteria when compared to untreated bacteria. To identify the molecular mechanisms involved in this phenomenon, we carried out a high-throughput genetic screen. A transposon library of *S*. Typhimurium mutants was subjected to negative selection in the presence of a sub-inhibitory concentration of CTX and genes related to anaerobic metabolism, biosynthesis of purines, pyrimidines, amino acids and other metabolites were identified as needed to survive in this condition. In addition, an impaired ability for oxygen consumption was observed when bacteria were cultured in the presence of a sub-inhibitory concentration of CTX. Altogether, our data indicate that exposure to sub-lethal concentrations of CTX increases the systemic colonization of *S.* Typhimurium in BALB/c mice in part by the establishment of a fitness alteration conducive to anaerobic metabolism.

## 1. Background

The inappropriate use of antibiotics for different human activities and the uncontrolled abuse of these compounds for therapeutic purposes has led to the constant emergence of clinically relevant resistant bacteria [1]. Moreover, during the last years, a dual, dose-dependent role for antibiotics has been proposed. Whereas at lethal doses these compounds kill or inhibit growth of most sensitive bacterial populations, sub-inhibitory concentrations mediate physiological changes, acting as signalling molecules [2]. These changes affect a broad range of processes and may generate fitness alterations as it has been recently reviewed [3]. A very interesting trait of this phenomenon is the ability of sub-inhibitory concentrations of antibiotics to modulate the expression of virulence-related processes such as adherence, quorum-sensing and motility among others (table 1). However, *in vivo* evidence supporting these findings is scarce.

**Table 1.** Changes in expression of virulence factors mediated by exposure to sub-inhibitory concentrations of antibiotics. MDR, multidrug resistant.

bacteria	antibiotic	effect on virulence	references
*S*. Typhimurium	tetracycline	induction of genes involved in regulation of SPI-1, iron uptake and acid tolerance and motility. Increased invasiveness of epithelial cells	[4]
*Pseudomonas aeruginosa*	azithromycin	decrease quorum-sensing, motility, synthesis of virulence factors and oxidative stress response. Induction of type III secretion system	[5]
*S*. Typhimurium	cationic peptides	induction of PhoP/PhoQ and RpoS virulence-related regulons. Repression of genes required for flagella synthesis and the invasion-associated type III secretion system	[6]
*S*. Typhimurium	amoxicillin, tetracycline	hypervirulence *in vitro* and *in vivo* (HEp2 cells and *C. elegans*, respectively)	[7]
MDR *S*. Typhimurium	tetracycline	accelerates invasiveness of epithelial cells	[8]
*Staphylococcus aureus*	cell wall-active antibiotics and fluoroquinolones	induction of virulence factors and quorum-sensing genes	[9]
*S. aureus*	cell wall-active antibiotics	induction of virulence genes	[10]
*Chromobacterium violaceum*	protein synthesis inhibitors	induction of quorum-sensing-related virulence factors. Induction of biofilm formation	[11]
*S*. Typhimurium	nalidixic acid	induction of SPI-2, PhoP/PhoQ, efflux pumps and peptidoglycan synthesis-related genes. Repression of SPI-1, lipopolysaccharide synthesis, motility-related and porins genes	[12]
*S. aureus*	ciprofloxacin and trimethoprim	induction of *recA* (SOS response), *Φ*13 phage-related genes and phage-encoded virulence staphylokinase (*sak*) gene	[13]
*P. aeruginosa* and *Proteus mirabilis*	mupirocin	reduction in flagellin expression which causes inhibition of formation of flagella and reduced bacterial motility	[14]

*Salmonella enterica* serovar Typhimurium (hereafter *S*. Typhimurium) is a facultative intracellular pathogen that causes systemic infection in mice and, most importantly, gastroenteritis in millions of people every year generating great economic losses. The molecular mechanisms of *S*. Typhimurium pathogenesis include two type-three secretion systems (T3SSs) encoded in *Salmonella* pathogenicity island 1 and 2 (SPI-1 and SPI-2, respectively). These secretion systems allow bacterial internalization and survival within eukaryotic cells, including macrophages [15,16]. To date, vast information related to the molecular mechanisms involved in *Salmonella* pathogenicity is available (reviewed in [17–19]). In contrast, the modulatory effect of sub-inhibitory concentrations of antibiotics on the virulence of this pathogen has not been explored and therefore it is worth evaluating.

In this study, we determined that exposure to a sub-inhibitory concentration of the third generation cephalosporin cefotaxime (CTX) increases the systemic colonization of *S*. Typhimurium in BALB/c mice. We conducted a series of genomic and biochemical assays that revealed the contribution of metabolic genes to the observed phenotype. Notably, we observed that a switch to anaerobic metabolism (generated by exposure to a sub-inhibitory concentration of CTX during aerobic growth) is required to increase the systemic colonization of *S*. Typhimurium in this animal model. These findings suggest another important issue in antibiotic treatment of infections, that it is linked not only to the emergence and selection of resistant strains in mismanaged therapies, but also to the development of more aggressive bacteria.

## 2. Material and methods

### 2.1. Bacterial strains and culture conditions

The *S.* Typhimurium strains used in this study are derivatives of the wild-type strain ATCC 14028s (table 2). Bacteria were grown routinely at 37°C with vigorous shaking in Luria-Bertani (LB) medium (10 g l^−1^ tryptone, 5 g l^−1^ yeast extract, 5 g l^−1^ NaCl). When required, the medium was supplemented with ampicillin (Amp; 100 µg ml^−1^), kanamycin (Kan; 75 µg ml^−1^) or chloramphenicol (Cam; 20 µg ml^−1^). Solid media included Bacto agar (15 g l^−1^).

**Table 2.** Strains used in this study.

strain	relevant genotype or characteristic	source
14028s	*S*. Typhimurium wild-type virulent strain	laboratory collection
Δ*phoN*::Kan	14028s Δ*STM4319*::Kan	laboratory collection
Δ*phoN*::Cam	14028s Δ*STM4319*::Cam	laboratory collection

### 2.2. Construction of mutant strains

Mutant strains with a deletion of the *phoN* gene and the concomitant insertion of a Kan- or Cam-resistance cassette were constructed using the Lambda Red recombination method, with modifications [20,21]. The presence of each mutation was confirmed by PCR amplification and then transferred to the wild-type genetic background by generalized transduction using phage P22 HT105/1 *int*-201. Table S1 of the electronic supplementary material includes the sequences of all primers used in this study.

### 2.3. Determination of minimal inhibitory concentration values

The minimal inhibitory concentration (MIC) of CTX for each bacterial strain was determined by microdilution in LB medium inoculated with 5 × 10^5^ CFU ml^−1^ and incubated at 37°C without agitation [22]. The lowest concentration of CTX that inhibited bacterial growth after 18 h of exposure was defined as the corresponding MIC.

### 2.4. Identification of genes required for cefotaxime survival *in vitro* by negative selection

A library containing approximately 60 000 mutants of *S*. Typhimurium 14028s harbouring random insertions of the EZ-Tn*5*<T7/KAN-2> transposon was kindly provided by Dr Michael McClleland (University of California Irvine, Irvine, CA, USA). A 1 : 1000 dilution of an overnight culture of the library was grown at 37°C in LB with aeration for 3 h. When OD_600_ reached 0.6, the culture was split in two independent flasks and CTX was added to a final concentration of 0.065 mg l^−1^ (0.5× MIC). Treated and untreated cultures were further incubated for 3 h at 37°C. Total DNA was extracted from treated and untreated samples from three independent experiments. Transposon insertions in genes required to survive to CTX *in vitro* were identified by competitive hybridizations using custom *Salmonella* genomic microrrays [20,23]. To do this, DNA from each sample was fragmented by sonication and polyA tails were added to the fragmented DNA using terminal transferase. Then, a nested PCR strategy was used to amplify only the polyA-tailed DNA fragments containing the transposon end carrying the P_T7_ and the genomic DNA adjacent to the insertion. An aliquot of each nested PCR reaction was used as template for a T7 *in vitro* transcription reaction. The RNA generated was used as template to synthesize labelled cDNA by incorporation of Cy5-dCTP (untreated samples) or Cy3-dCTP (CTX-treated samples) using reverse transcriptase. Finally, labelled cDNA from CTX-treated and untreated samples was mixed in equal amounts and hybridized in slides containing a *Salmonella* microarray printed in triplicate [20,23]. Hybridized chips were scanned using a ScanArray GX (Perkin Elmer) scanner and images were analysed using GenePix Pro v. 6.0 software. Data were normalized and analysed using Webarray (www.webarraydb.org) [24], with quantile normalization. Mutants exhibiting a log_2_-fold change ratio (*M*) value of less than or equal to −0.75, a signal intensity (*A*) value of greater than or equal to 8 and a *p*-value of less than or equal to 0.005 were considered as negatively selected *in vitro*.

### 2.5. Animal studies

Fresh LB media were inoculated in a 1 : 1000 dilution with an overnight culture of *S*. Typhimurium Δ*phoN*::Kan and Δ*phoN*::Cam in independent flasks and incubated at 37°C with agitation. When OD_600_ reached 0.6, each culture was split in two independent flasks and CTX was added to a final concentration of 0.065 mg l^−1^ (0.5× MIC) in one of them. Treated and untreated cultures were further incubated for 3 h at 37°C with or without agitation, as required to assess the effect of CTX under aerobic or anaerobic conditions. Then, an aliquot of each culture was rinsed with sterile PBS to eliminate the antibiotic and used to determine CFU counts by serial dilution and plating on LB agar plates.

To evaluate the effect of CTX in *S*. Typhimurium systemic colonization, groups of five BALB/c mice (6–8 weeks old, female) were inoculated intraperitoneally (IP) with approximately 2 × 10^5^ CFU of a 1 : 1 mixture of CTX-exposed and non-exposed (control) bacterial cultures. After 48 h of infection, mice were euthanized; the spleens and livers were removed aseptically and homogenized in 5 ml of sterile ice-cold PBS using a T25 digital ULTRA-TURRAX (IKA). Aliquots of each homogenized organ were serially diluted in sterile PBS and plated on LB agar plates to assess CFU counts and the competitive index (CI), as described [23].

### 2.6. Oxygen consumption

*S*. Typhimurium 14028s was grown in the presence or absence of CTX (0.065 mg l^−1^; 0.5× MIC), as described above. Aliquots of 2 ml from treated and untreated cultures were diluted fivefold in fresh LB medium and 2 ml of sterile mineral oil was added on the surface to prevent oxygen diffusion. Oxygen consumption was determined using a Fibox III oxygenmeter equipped with an oxygen-sensitive probe and an optic fibre reader (Presens). Results were expressed as percentage of oxygen consumed during an 8 min period and normalized by protein concentration [25].

## 3. Results

### 3.1. A sub-inhibitory concentration of cefotaxime increases the systemic colonization of *S*. Typhimurium in the murine model

Little is known about the antibiotic-mediated modulation of virulence in *Salmonella*. To evaluate the effect of a sub-inhibitory concentration of CTX on the systemic colonization of *S*. Typhimurium, we first determined the MIC of CTX for the wild-type strain14028s and the Δ*phoN*::Kan and Δ*phoN*::Cam derivatives by the microdilution test. We decided to use Δ*phoN* mutants because it has been shown that the deletion of the *phoN* gene does not impact the colonization abilities of *S.* Typhimurium in the mice model [26]. Therefore, using the selectable markers associated with these mutants we can monitor full-virulent isogenic strains grown in the presence or absence of a sub-inhibitory concentration of CTX.

The MIC of CTX for strain 14028s and the Δ*phoN*::Kan and Δ*phoN*::Cam mutants was 0.13 mg l^−1^. To assess cell viability of these strains when exposed to a sub-inhibitory concentration of the antibiotic, bacteria were grown in the presence or absence of 0.5× MIC of CTX (0.065 mg l^−1^); a five- to sixfold decrease in CFU counts in CTX-exposed cultures was observed accompanied by signs of cell lysis (data not shown).

**Figure 1.**
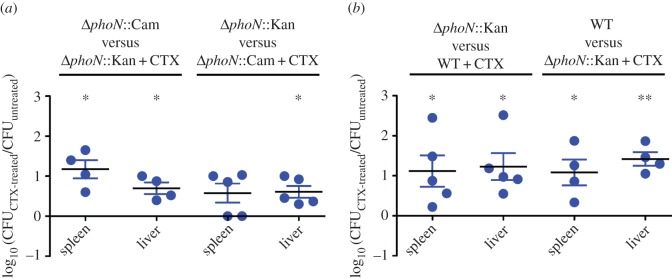
Increased systemic colonization of *S*. Typhimurium exposed to a sub-inhibitory concentration of CTX during aerobic growth. Groups of five BALB/c mice were inoculated IP with (*a*) a 1 : 1 mixture of the Δ*phoN*::Kan mutant grown in the presence of CTX (0.065 mg l^−1^; 0.5× MIC) and the Δ*phoN*::Cam mutant grown in the absence of the antibiotic or the reciprocal combination, or (*b*) a 1 : 1 mixture of the untreated Δ*phoN*::Kan and the wild-type (WT) strain grown in the presence of CTX (0.065 mg l^−1^; 0.5× MIC) or the reciprocal combination. After 2 days of infection, mice were euthanized and the liver and spleen were aseptically removed and homogenized in sterile PBS. Bacterial load recovered from each organ was determined by plating serial 10-fold dilutions on LB agar with the appropriate antibiotics. CI values were calculated as a mean ratio of CTX-treated to untreated control, normalized to the input ratio and converted logarithmically. Error bars denote standard error. Statistical significance was determined using a two-tailed Student's *t*-test. Asterisks indicate normalized output ratios that were significantly statistically different from zero, the ideal value obtained when both strains colonize to the same extent (*p < 0.05; **p < 0.01).

To determine the effect of a sub-inhibitory concentration of CTX on the colonization ability of this pathogen, the CI for treated and untreated bacteria was evaluated in BALB/c mice. Briefly, Δ*phoN*::Kan and Δ*phoN*::Cam mutants were grown *in vitro* and incubated for 3 h in the presence or absence of CTX (0.065 mg l^−1^; 0.5× MIC). Then, a 1 : 1 mixture of treated and untreated bacteria was injected IP in groups of BALB/c mice. After 48 h of infection, an increased colonization of internal organs (liver and spleen) was observed for CTX-exposed bacteria compared to untreated bacteria in both derivative strains (figure 1*a*). This was also observed when the experiment was repeated using the Δ*phoN*::Kan mutant and the wild-type strain, indicating that the effect of CTX exposure is not related to the lack of the *phoN* gene (figure 1*b*). Thus, these results indicate that growth in the presence of a sub-inhibitory concentration of CTX (0.5× MIC) increases the systemic colonization of *S*. Typhimurium in mice.

### 3.2. Identification of genes required to survive in the presence of a sub-inhibitory concentration of cefotaxime *in vitro*

**Figure 2.**
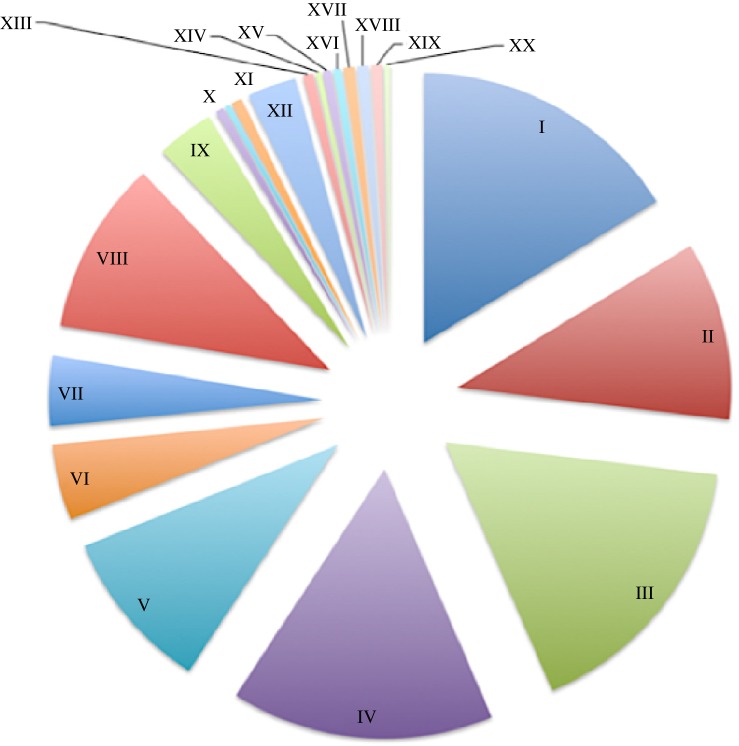
GO classification of mutants under negative selection *in vitro*. Using GO terms, we classified by functional categories the percentage of mutants under negative selection generated as a consequence of CTX exposure. Our analysis showed that mutants under selection were grouped in the following categories: (I) cellular-metabolic, (II) single-organism metabolic, (III) organic substance metabolic, (IV) primary metabolic, (V) biosynthetic, (VI) single-organism cellular processes, (VII) establishment of localization, (VIII) nitrogen compound metabolic, (IX) regulation of biological processes, (X) single-organism signalling, (XI) pathogenesis, (XII) methylation, (XIII) catabolic process, (XIV) regulation of biological quality, (XV) response to chemical stimulus, (XVI) localization of cell, (XVII) cellular component organization, (XVIII) cellular component biogenesis, (XIX) response to stress, (XX) macromolecule localization and (XXI) response to external stimulus.

To identify genes and biological pathways required to survive in the presence of the same concentration of CTX shown to increase the colonization in mice, a transposon library containing approximately 60 000 mutants of *S*. Typhimurium 14028s was grown *in vitro* in the presence and absence of CTX (0.065 mg l^−1^; 0.5× MIC). This growth condition is responsible for the metabolic state of bacteria prior to mice inoculation in our competition assays. Mutants under negative selection *in vitro* in the presence of CTX were identified by means of a high-throughput genetic screen. The analysis of our data showed that mutants in 263 genes are defective for *in vitro* growth in the presence of CTX (electronic supplementary material, table S2). Therefore, these mutants lack genes that are required to survive the damage generated by the exposure to the antibiotic *in vitro*. These genes were classified by functional categories using Gene Ontology (GO) terms. Thus, mutants in genes belonging to GO categories (i) organic substances metabolism, (ii) primary metabolic processes, and (iii) biosynthetic metabolism of different metabolites are lost from the population after CTX exposure, suggesting that genes related to those processes are required to survive in the presence of a sub-inhibitory concentration of CTX (figure 2).

We also identified another set of mutants corresponding to genes related to (i) membrane/periplasmic proteins, (ii) stress–response proteins and (iii) anaerobic metabolism (50, 16 and 20 mutants, respectively) (electronic supplementary material, table S2). In addition, we determined that mutants in a few genes encoding known virulence factors are under negative selection after exposure to CTX (figure 2) as well. Among these, we found mutants in genes related to the biosynthesis of fimbriae (*safD*, *fimZ*, *pefB*, *sthA*, *stfA*), flagella (*fliC*, *fliN*, *flgG*) and lipopolysaccharide (*rfaH*, *wzzB*).

### 3.3. Identification of a common set of genes required to survive exposure to cefotaxime *in vitro* and for systemic colonization *in vivo*

Previous data from our laboratory using a similar transposon library in *S*. Typhimurium 14028s allowed the identification of genes required for systemic colonization of BALB/c mice [27]. Thus, we compared the dataset from this study with the database from the negative selection *in vitro* after exposure to CTX (0.065 mg l^−1^; 0.5× MIC). This analysis revealed 23 genes required for systemic colonization in mice and for survival in the presence of a sub-inhibitory concentration of CTX *in vitro* (table 3). Since many of these genes encode hypothetical proteins, we were only able to obtain biologically relevant information for *folK*, *fbaB*, *glgP*, *hisA*, *pyrE*, *thiL* and *rluC* genes, encoding proteins that participate in the metabolism of purines, pyrimidines, amino acids and secondary metabolites. In addition, we identified the *caiB*, *STM1496* and *yedY* genes, which encode proteins that have a role in energy generation during anaerobic growth. Finally, genes *trg*, *yhjE* and *fimZ* that encode proteins involved in aspartate and maltose chemotaxis, transport of metabolites inside the cell and regulation of bacterial fimbriae and flagella production, respectively, were also detected (table 3).

**Table 3.** Mutants under negative selection in the presence of a sub-inhibitory concentration of CTX *in vitro* that are impaired for systemic colonization of BALB/c mice.

gene number	gene symbol	function
*STM3524*	*glpG*	protein of *glp* regulon
*STM0183*	*folK*	7,8-dihydro-6-hydroxymethylpterin-pyrophosphokinase, PPPK
*STM2141*	*fbaB*	3-oxoacyl-[acyl-carrier-protein] synthase I (fructose-bisphosphate aldolase)
*STM2385*	*yfcB*	putative methylase
*STM3119*		putative monoamine oxidase
*STM3534*	*glgP*	glycogen phosphorylase
*STM2076*	*hisA*	N-(5′-phospho-L-ribosyl-formimino)-5-amino-1-(5′-phosphoribosyl)-4-imidazolecarboxamide isomerase
*STM14_0085*	*caiB*	l-carnitine dehydratase
*STM0549*	*fimZ*	fimbrial protein Z
*STM3733*	*pyrE*	orotate phosphoribosyltransferase
*STM1496*		putative dimethylsulfoxide reductase
*STM1190*	*yceD*	putative metal-binding
*STM3609*	*yhjE*	putative MFS family transport protein
*STM0419*	*thiL*	thiamin-monophosphate kinase
*STM4010*		putative hydrolase
*STM3377*		putative nitrate reductase
*STM1793*		putative cytochrome oxidase, subunit II
*STM1626*	*trg*	methyl-accepting chemotaxis protein III, ribose and galactose sensor receptor
*STM1187*	*rluC*	23S rRNA pseudouridylate synthase
*STM0765*		putative cation transporter
*STM3833*		putative mandelate racemase/muconate lactonizing enzyme family
*STM0565*		putative periplasmic protein
*STM1931*	*araH*	putative intracellular protease/amidase

### 3.4. Exposure to a sub-inhibitory concentration of cefotaxime during aerobic growth induces a switch to anaerobic metabolism

**Figure 3.**
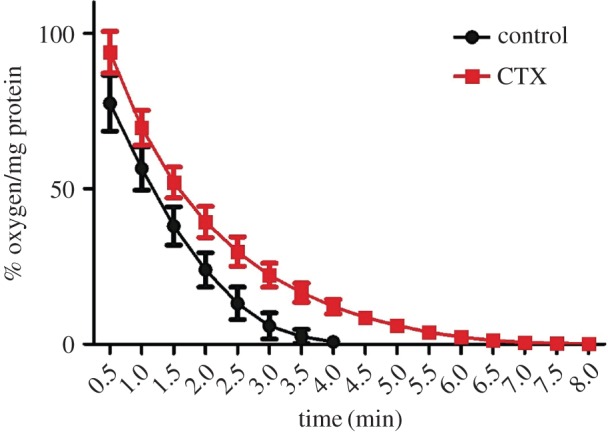
CTX affects the ability to consume oxygen in *S*. Typhimurium. Bacterial cultures of *S.* Typhimurium 14028s grown in the presence or absence of CTX (0.065 mg l^−1^; 0.5× MIC) were assayed for its ability to consume oxygen. Plot shows values of oxygen available normalized by protein content of the sample and represent the mean of three independent trials. Error bars denote standard error.

Our genetic screen suggested a strong contribution of metabolic genes to survive the stress generated by a sub-inhibitory concentration of CTX (0.5× MIC) *in vitro* that could be responsible for the phenotype observed in mice (electronic supplementary material, table S2, and table 3, respectively). Since we identified genes related to anaerobic metabolism as required to survive *in vitro* exposure to CTX, and considering that anaerobiosis contributes to *Salmonella* virulence [28–31], we decided to validate our predictions by evaluating changes in the bacterial oxygen consumption after treatment with the antibiotic. We determined that after 3 h of exposure to a sub-inhibitory concentration of CTX (0.5× MIC), the bacterial ability to consume oxygen is impaired and delayed compared with an untreated control (figure 3). These results agree with the data from our genetic screen and suggest that the presence of CTX causes a metabolic switch to anaerobic metabolism, even when *S*. Typhimurium is incubated aerobically.

Microarray-based global transcriptional profiling experiments of *S*. Typhimurium cultures exposed for 3 h to the same sub-inhibitory concentration of CTX as that associated with an increased colonization of mice (0.5× MIC) showed no changes in the expression of traditional anaerobiosis-related genes (data not shown). However, it is possible that bacterial transcriptional response associated with the metabolic switch takes place at earlier exposure times. In addition, the analysis of our transcriptomic data also indicates that the presence of a sub-inhibitory concentration of CTX during aerobic growth does not affect the *in vitro* expression profile of key genes linked to *Salmonella* virulence (e.g. SPI-1 and SPI-2) (data not shown).

**Figure 4.**
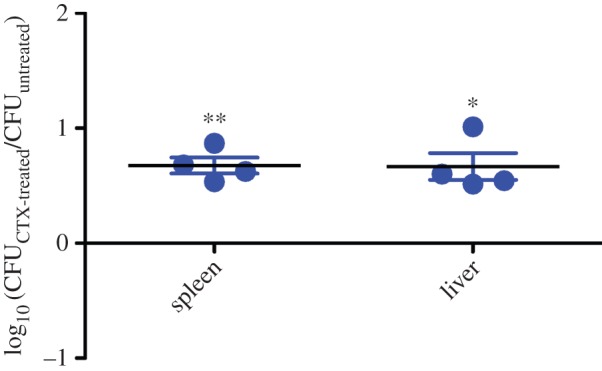
Increased systemic colonization of *S*. Typhimurium exposed to a sub-inhibitory concentration of CTX during anaerobic growth. Groups of five BALB/c mice were inoculated IP with a 1 : 1 mixture of the Δ*phoN*::Kan mutant grown in the presence of CTX (0.065 mg l^−1^; 0.5× MIC) and the Δ*phoN*::Cam mutant grown in the absence of the antibiotic. After 2 days of infection, mice were euthanized and the liver and spleen were aseptically removed and homogenized in sterile PBS. Bacterial load recovered from each organ was determined by plating serial 10-fold dilutions on LB agar plates with the appropriate antibiotics. CI values were calculated as a mean ratio of CTX-treated to untreated control, normalized to the input ratio and converted logarithmically. Error bars denote standard error. Statistical significance was determined using a two-tailed Student's *t*-test. Asterisks indicate normalized output ratios that were significantly statistically different from zero, the ideal value obtained when both strains colonize to the same extent (*p < 0.05; **p < 0.01).

On the other hand, it has been described that bactericidal antibiotics, and specifically CTX, generate oxidative stress [32–34]. However, this concept remains controversial as many publications have challenged the original findings [35,36]. To evaluate if colonization mediated by CTX is a consequence of the bacterial response to oxidative stress, we assessed the ability of *S*. Typhimurium cultures exposed to the antibiotic in anaerobic conditions to colonize BALB/c mice systemically. Since bacteria were exposed to antibiotic in the absence of oxygen, no formation of reactive oxygen species was expected. Antibiotic-exposed bacteria grown under anaerobic conditions showed an increased colonization of liver and spleen compared to untreated bacteria (figure 4), indicating that oxidative stress mediated by CTX is not responsible for the observed phenotype.

## 4. Discussion

It has been shown that some genes encoding virulence factors in different bacterial models can be induced after exposure to sub-inhibitory concentrations of several antibiotics (table 1). However, *in vivo* evidence showing that antibiotics could increase bacterial virulence is scarce to date. In this context, it has recently been shown that ciprofloxacin promotes bacterial virulence by generating subpopulations of virulent and avirulent phenotypes in a bi-stable fashion. This phenomenon is called ‘cooperative virulence’ and might increase bacterial transmission in a mouse model for *Salmonella* diarrhoea [37,38]. These findings strongly agree with our results in a murine model of infection where an increased systemic colonization of *S*. Typhimurium is observed after exposure to CTX (figure 1). As mentioned, controversial evidence has been shown indicating that bactericidal antibiotics owe part of their toxicity to oxidative stress establishment [33,34]. Notably, the colonization phenotype is not a consequence of bacterial adaptation to the oxidative damage generated by the antibiotic (figure 4), as has been shown previously [32].

To understand this phenotype, we based our assays on the idea that *S*. Typhimurium requires a set of biological functions to cope with the damage generated by the exposure to a sub-inhibitory concentration of antibiotic. These fitness alterations would be responsible for the colonization phenotype and, most probably, the consequence of an adaptation to the presence of the antibiotic. To identify processes or biological targets required to survive exposure to CTX *in vitro*, we used a high-throughput genetic screen to identify mutants under negative selection [23]. We used a transposon-insertion mutant library and competitive DNA hybridizations on *Salmonella* genomic microarrays to identify mutants that are lost from the population under selection after antibiotic treatment. Using this genomic approach, we identified genes required to cope with CTX-mediated stress *in vitro*. Surprisingly, an important metabolic component was observed, suggesting a change in the bacterial physiology in response to antibiotic exposure (figure 2). Among these, we found mutants in genes encoding membrane/periplasmic and different stress-response proteins (electronic supplementary material, table S2). These results were expected because the canonical target of CTX is the bacterial cell wall and, also, this antibiotic generates oxidative stress which damages DNA and proteins as previously reported [32].

In addition, the comparison of data on mutants under negative selection *in vitro* after CTX exposure with previous data generated in our laboratory on mutants under negative selection *in vivo* during systemic colonization of BALB/c mice allowed us to identify a common set of genes that participate in the biosynthesis of purines, pyrimidines, amino acids and secondary metabolites (table 3), suggesting that these genes are needed for the antibiotic-mediated enhanced colonization of *Salmonella*. This is in agreement with a recent report describing that genes involved in the mentioned processes are negatively selected during *Yersinia pestis* colonization of deep tissue in mice [39]. Similarly, it has been reported that biosynthesis of asparagine, tryptophan and glycine is required for *S*. Typhimurium pathogenesis [40] and that genes involved in biosynthesis of nucleotides are critical for growth of this pathogen in human blood [41]. Although we did not observe the same genes in our study, we still believe that these processes are required for CTX-mediated enhanced colonization in *S*. Typhimurium since genes *folK*, *fbaB*, *glgP*, *hisA*, *pyrE*, *thiL* and *rluC* encode proteins required to synthesize precursors of these molecules (table 3).

Our genetic screen also allowed us to determine that mutants in genes *glpG*, *atpH*, *plsB*, *fabA*, *narZ*, *selB*, *gudT*, *metH*, *nuoA*, *caiB*, *tktB*, *cbiA*, *STM1496*, *msbB*, *ttrC*, *STM3377*, *ylbA*, *yjfM*, *dmsA* and *eutE* are under negative selection when exposed to CTX *in vitro* (electronic supplementary material, table S2). It has been suggested that all these genes play a role in the anaerobic metabolism, allowing the use of alternative electron acceptors such as nitrate and tetrathionate [42,43] or alternative carbon sources such as ethanolamine and carnitine [44,45] in this condition.

The diminished oxygen consumption observed in antibiotic-exposed cultures (figure 3) supports our findings and suggests that a metabolic switch to anaerobic metabolism is induced by CTX, even when oxygen is available during the antibiotic treatment. This response seems to represent a global defence mechanism against different classes of stress since it has been also observed as a consequence of exposure to metals, high salt concentrations and phage infection in different bacterial models [25,46,47]. Another non-exclusive possibility is that the anaerobic switch mediated by CTX might involve the cooperative virulence phenomenon which is determined by persister cells [37,38]. This subpopulation is highly tolerant to antibiotics and its formation has been related to the *glp* regulon and the *plsB* gene [48]. Both of them were identified in our screens as genes required to survive in the presence of CTX (electronic supplementary material, table S2).

Altogether, our results suggest that the increased systemic colonization of *S.* Typhimurium in mice after CTX exposure is mediated, at least in part, by a metabolic adaptation and switch to anaerobic metabolism and biosynthesis of essential metabolites. CTX might predispose bacterial physiology to be better adapted to the intestinal environment where oxygen availability is scarce [49], since when administered by the IP route the gastric phase of infection is avoided and bacteria can rapidly re-circulate to occupy the gut. These results show a fitness alteration allowing bacteria to be pre-adapted to a completely different ecologic niche or selecting pressure, which represents a bacterial strategy to generate infection, a phenomenon known as ‘adaptive prediction’ [50]. Finally, it is worth mentioning that this study represents an alert and reinforces the value in regulating the indiscriminate use of antibiotics, due to the risk of not only selecting new multi-resistant bacteria but also the generation of more aggressive strains.

## Supplementary Material

150909_OB_RMolina_Supplementary material.doc

## Data accessibility

Microarray hybridization data are deposited as GSE61988 series in Gene Expression Omnibus (GEO) at the NCBI's database.
