# Supplementary material for: Exposure to sub-inhibitory concentrations of cefotaxime enhances the systemic colonization of Salmonella Typhimurium in BALB/c mice
Source: Open Biol. 2015 Oct 14;5(10):150070. doi: 10.1098/rsob.150070 (PMC4632510; doi:10.1098/rsob.150070)
Supplement: 150909_OB_RMolina_Supplementary material.doc [file rsob150070supp1.doc]

**Exposure to sub-inhibitory concentrations of cefotaxime enhances the systemic colonization of *Salmonella* Typhimurium in BALB/c mice**

Roberto C. Molina-Quiroz^1,2^**^#^**, Cecilia A. Silva^1^, Cristian F. Molina^3^, Lorenzo E. Leiva^4^, Sebastián Reyes-Cerpa^5^, Inés Contreras^1^, and Carlos A. Santiviago^1^**^#^**

^1^Laboratorio de Microbiología, Departamento de Bioquímica y Biología Molecular, Facultad de Ciencias Químicas y Farmacéuticas, Universidad de Chile, Santiago, Chile. ^2^Center for Adaptation Genetics and Drugs Resistance, Molecular Biology and Microbiology Faculty, Tufts University, Boston, USA. ^3^AUSTRAL-Omics, Universidad Austral de Chile, Valdivia, Chile. ^4^Centro de InmunoBioTecnología, Programa Disciplinario de Inmunología, Instituto de Ciencias Biomédicas, Universidad de Chile, Santiago, Chile. ^5^Laboratorio de Virología, Centro de Biotecnología Acuícola (CBA), Facultad de Química y Biología, Universidad de Santiago de Chile, Santiago, Chile.

**^#^** Corresponding authors.

**Supplementary material:**

**Table S1.** Primers used in this study.

**Table S2.** Identification of mutants under negative selection in *S*. Typhimurium cultures exposed to a sub-lethal concentration of CTX *in vitro*.

**Table S1. Primers used in this study.**

| **Primer** | **Sequence** |
| --- | --- |
| STM_∆*phoN*_(H1+P1) | GTGAGTCTTTATGAAAAGTCGTTATTTAGTATTTTTTCTAGTGTAGGCTGGAGCTGCTTC |
| STM_∆*phoN*_(H2+P2) | ACTTTCACCTTCAGTAATTAAGTTCGGGGTGATCTTCTTTCATATGAATATCCTCCTTAG |
| STM_∆*phoN*_OUT5 | TTGCCTGATCCGGAGTGA |
| K1 | CAGTCATAGCCGAATAGCCT |
| C3 | CAGCTGAACGGTCTGGTTATAGG |

**Table S2. Identification of mutants under negative selection in *S*. Typhimurium cultures exposed to a sub-lethal concentration of CTX *in vitro*.**

A library of ~60.000 EZ-Tn*5* <T7/KAN-2> transposon insertion mutants in *S*. Typhimurium 14028s was incubated in the presence and absence of CTX (0.065 mg/L; 0.5x MIC). Mutants under negative selection *in vitro* were identified by competitive hybridizations in *Salmonella* genomic microarrays.

| **Gene number** | **Gene symbol** | **Function** |
| --- | --- | --- |
| *STM0947* |  | putative integrase protein |
| *STM1708* | *yciM* | putative N-acetylglucosaminyl transferase |
| *STM2605* |  | Gifsy-1 prophage: similar to head-tail preconnector gp5 of phage 21 |
| *STM3524* | *glpG* | protein of glp regulon |
| *STM3450* | *yheM* | putative oxidation of intracellular sulfur |
| *STM3528* |  | putative periplasmic phosphate-binding protein |
| *STM0392* | *rdgC* | putative exonuclease involved in removal of stalled replication fork |
| *STM3868* | *atpH* | membrane-bound ATP synthase, F1 sector, delta-subunit |
| *STM3178* | *ygiY* | putative sensory histidine kinase in regulatory system |
| *STM1159* | *yceO* | putative inner membrane protein |
| *STM0302* | *safD* | putative fimbriae subunit |
| *STM1297* | *selD* | selenophosphate synthase |
| *STM3559* | *yhhV* | putative cytoplasmic protein |
| *STM2795* | *ygaU* | putative LysM domain |
| *PSLT004* | *repA3* | DNA replication |
| *STM4235* | *plsB* | glycerolphosphate acyltransferase activity |
| *STM0971* |  | putative cytoplasmic protein |
| *STM4459* | *pyrI* | aspartate carbamoyltransferase, regulatory subunit (allosteric regulation) |
| *STM0872* | *grxA* | glutaredoxin1 redox coenzyme for glutathione-dependent ribonucleotide reductase |
| *STM2838.S* | *gutQ* | putative sugar phosphate isomerase |
| *STM3012* |  | putative transcriptional regulator |
| *STM0897* |  | Fels-1 prophage |
| *STM1067* | *fabA* | beta-hydroxydecanoyl thioester dehydrase (trans-2-decenoyl-ACP isomerase) |
| *STM2392* | *vacJ* | lipoprotein precursor |
| *STM0349* |  | putative outer membrane lipoprotein |
| *STM1577* | *narZ* | nitrate reductase 2, alpha subunit |
| *STM3091* | *galP* | MFS family, galactose:proton symporter |
| *STM2983* | *ygdI* | putative lipoprotein |
| *STM2850* | *hycD* | hydrogenase 3, membrane subunit (part of FHL complex) |
| *STM0362* |  | putative cytoplasmic protein |
| *STM0042* |  | putative sodium galactoside symporter |
| *STM0183* | *folK* | 7,8-dihydro-6-hydroxymethylpterin-pyrophosphokinase, PPPK |
| *STM3031* |  | ail and ompX homologue |
| *STM1653* |  | putative membrane transporter of cations |
| *STM4472* | *ytgA* | putative inner membrane protein |
| *STM3682* | *selB* | selenocysteinyl-tRNA-specific translation factor |
| *STM0688* | *ybfN* | putative lipoprotein |
| *STM0217* | *tsf* | protein chain elongation factor EF-Ts |
| *STM3541* | *gntU* | low affinity gluconate permease |
| *STM3184* | *yqiB* | putative cytoplasmic protein |
| *STM1856* |  | putative cytoplasmic protein |
| *STM2386* | *yfcN* | putative Smr domain |
| *STM1083* | *yccX* | putative phosphohydrolase |
| *STM2141* | *fbaB* | 3-oxoacyl-[acyl-carrier-protein] synthase I |
| *STM2597* |  | Gifsy-1 prophage: similar to major tail protein |
| *STM2385* | *yfcB* | putative methylase |
| *STM1768* | *ychP* | putative invasin |
| *STM3119* |  | putative monoamine oxidase |
| *STM4597* |  | putative periplasmic protein |
| *STM0745* | *tolQ* | inner membrane proteins required for outer membrane integrity |
| *STM3534* | *glgP* | glycogen phosphorylase |
| *STM3642* | *tag* | 3-methyl-adenine DNA glycosylase I, constitutive |
| *STM3767* |  | putative cytoplasmic protein |
| *STM4253* |  | putative outer membrane lipoprotein |
| *STM3480* | *yhfL* | putative outer membrane lipoprotein |
| *STM4060* | *cpxP* | periplasmic repressor of cpx regulon by interaction with CpxA, rescue from transitory stresses |
| *STM3424* | *rplR* | 50S ribosomal subunit protein L18 |
| *STM2680* |  | putative cytoplasmic protein |
| *STM1898* | *ruvC* | Holliday junction nuclease |
| *STM2962* | *gudT* | putative MFS superfamily, D-glucarate permease |
| *STM2076* | *hisA* | N-(5'-phospho-L-ribosyl-formimino)-5-amino-1-(5'-phosphoribosyl)-4-imidazolecarboxamide isomerase |
| *STM1677* |  | putative transcriptional regulator, LysR family |
| *STM4188.S* | *metH* | B12-dependent homocysteine-N5-methyltetrahydrofolate transmethylase |
| *STM2741* |  | putative periplasmic protein |
| *STM2328* | *nuoA* | NADH dehydrogenase I chain A |
| *STM0072* | *caiB* | l-carnitine dehydratase |
| *STM1271* | *yeaR* | putative cytoplasmic protein |
| *STM2474* | *tktB* | transketolase 2, isozyme |
| *STM1378* | *pykF* | pyruvate kinase I (formerly F), fructose stimulated |
| *STM0046* | *ileS* | isoleucine tRNA synthetase |
| *STM2491* | *bcp* | thiol peroxidase, thioredoxin dependent |
| *STM0549* | *fimZ* | fimbrial protein Z, putative transcriptional regulator (LuxR/UhpA family) |
| *STM0208* | *dgt* | deoxyguanosine triphosphate triphosphohydrolase |
| *STM4296* | *adi* | arginine decarboxylase, catabolic; inducible by acid |
| *STM2865* | *avrA* | putative inner membrane protein |
| *STM0559* | *rfbI* | putative glycosyl translocase |
| *STM1901* | *aspS* | aspartate tRNA synthetase |
| *STM1470* | *tus* | replication termination protein |
| *STM2508* |  | putative cytoplasmic protein |
| *STM3794* |  | putative regulatory protein, deoR family |
| *STM4464* |  | putative arginine repressor |
| *STM4365* | *yjeT* | putative inner membrane protein |
| *PSLT019* | *pefB* | plasmid-encoded fimbriae;regulation |
| *STM3294* | *mrsA* | phosphoglucosamine mutase |
| *STM2978* | *fucU* | conserved protein of fucose operon |
| *STM2035* | *cbiA* | synthesis of vitamin B12 adenosyl cobalamide precursor |
| *STM3438* | *rplW* | 50S ribosomal subunit protein L23 |
| *STM4567* | *deoC* | 2-deoxyribose-5-phosphate aldolase |
| *STM2195* |  | putative transcriptional regulator |
| *STM0852* | *yliG* | putative Fe-S oxidoreductases family 1 |
| *STM1201* | *holB* | DNA polymerase III, delta prime subunit |
| *STM1849* |  | putative inner membrane protein |
| *STM3641* | *yhjY* | putative lipase |
| *STM4322* | *yjdC* | putative bacterial regulatory protein, merR family |
| *STM3733* | *pyrE* | orotate phosphoribosyltransferase |
| *STM3164* | *yqhD* | putative alcohol dehydrogenase |
| *STM0742* | *ybgT* | putative outer membrane lipoprotein |
| *STM1077* | *yccT* | putative periplasmic protein |
| *STM2129* | *yegB* | putative MFS family transport protein |
| *STM3434* | *rpsC* | 30S ribosomal subunit protein S3 |
| *STM0817* | *ybhF* | putative ABC-type multidrug transport system, ATPase component |
| *STM1496* |  | putative dimethylsulfoxide reductase |
| *STM0120* | *yabC* | putative S-adenosyl methionine adenyltransferase |
| *STM2157* | *yehS* | putative cytoplasmic protein |
| *STM1909* | *argS* | arginine tRNA synthetase |
| *STM0400* | *proY* | putative APC family, proline transporter |
| *STM1223* | *potC* | ABC superfamily (membrane), spermidine/putrescine transporter |
| *STM1982* | *rcsA* | positive transcriptional regulator of capsular/exo- polysaccharide synthesis (LuxR/UhpA family) |
| *STM0502* | *ybbL* | putative ABC-type sugar/spermidine/putrescine transport system, ATPase component |
| *STM1889* | *msbB* | myristoyl transferase in lipid A biosynthesis, suppressor of htrB (lpxL) |
| *STM4240* | *yjbJ* | putative cytoplasmic protein |
| *STM3735* | *yicC* | putative stress-induced protein |
| *STM2395* | *pgtE* | Phosphoglycerate transport: outer membrane protein E |
| *STM0009* | *yaaH* | putative regulator |
| *STM1190* | *yceD* | putative metal-binding |
| *STM1286* | *mipA* | scaffolding protein for murein-synthesizing holoenzyme |
| *STM3935* | *hemY* | a late step of protoheme IX synthesis |
| *STM2811* | *proX* | ABC superfamily (bind_prot), glycine/betaine/proline transport protein |
| *STM3272* | *yhbS* | putative ABC superfamily (membrane) transport protein |
| *STM1746.S* | *oppA* | oligopeptide transport protein |
| *STM0651* |  | putative permease |
| *STM3903* | *ilvE* | branched-chain amino-acid aminotransferase |
| *STM0403* | *yajB* | putative cytoplasmic protein |
| *STM2368* | *truA* | pseudouridylate synthase I |
| *STM1460* | *ydgK* | putative inner membrane protein |
| *STM2935* | *cysD* | ATP-sulfurylase, subunit 1 (ATP:sulfate adenylyltransferase) |
| *STM3030* |  | putative periplasmic protein |
| *STM1993* | *yedJ* | putative hydrolase |
| *STM0821* | *dinG* | LexA regulated (SOS) repair enzyme |
| *STM2611.S* | *-* | endopeptidase-like protein |
| *STM2312* | *elaA* | putative acyltransferase |
| *STM2595* |  | Gifsy-1 prophage: similar to minor tail protein |
| *STM2820* | *yqaB* | putative phosphoglucomutase |
| *STM1771* | *chaA* | CaCA family, sodium-calcium/proton antiporter |
| *STM3609* | *yhjE* | putative MFS family transport protein |
| *STM2087* | *rfbV* | LPS side chain defect: abequosyltransferase |
| *STM2496* | *yfgE* | putative ATPase involved in DNA replication initiation |
| *STM3668* | *yiaK* | putative malate dehydrogenase |
| *STM3226* | *yqjA* | putative DedA family, membrane protein |
| *STM0419* | *thiL* | thiamin-monophosphate kinase |
| *STM1384* | *ttrC* | Tetrathionate reductase complex, subunit C |
| *STM4390* |  | putative cytoplasmic protein |
| *STM3750* | *yicJ* | putative GPH family transport protein |
| *STM3002* | *lgt* | phosphatidylglycerol-prolipoprotein diacylglyceryl transferase |
| *STM0859* |  | putative transcriptional regulator, LysR family |
| *STM0647* | *rlpB* | a minor lipoprotein |
| *STM2652* | *pssA* | phosphatidylserine synthase (CDP-diacylglycerol-serine O-phosphatidyltransferase) |
| *STM1654* | *ydaO* | putative ATPase |
| *STM0712* | *ybgJ* | putative carboxylase |
| *STM4299* | *melB* | GPH family, melibiose permease II |
| *STM0103* | *araB* | L-ribulokinase |
| *STM0907* |  | Fels-1 prophage; putative chitinase |
| *STM0649.S* | *-* | putative hydrolase |
| *STM3729* | *radC* | putative DNA repair protein, associated with replication forks |
| *STM1044* | *sodC* | Gifsy-2 prophage: superoxide dismutase precursor (Cu-Zn) |
| *STM2115* | *wcaA* | putative glycosyl transferase in colanic acid biosynthesis |
| *STM2505* |  | putative inner membrane protein |
| *STM3381* | *yhdT* | putative inner membrane protein |
| *STM3458* | *yheR* | putative NAD(P)H oxidoreductase |
| *STM0896* |  | Fels-1 prophage |
| *STM0141* | *guaC* | GMP reductase |
| *STM1959* | *fliC* | flagellar biosynthesis; flagellin, filament structural protein |
| *STM3440* | *rplC* | 50S ribosomal subunit protein L3 |
| *STM0717* |  | putative inner membrane protein |
| *STM0469* | *rpmE2* | putative 50S ribosomal protein L31 (second copy) |
| *STM4010* |  | putative hydrolase |
| *STM0102* | *araA* | L-arabinose isomerase |
| *STM1859* |  | putative cytoplasmic protein |
| *STM3555* | *ugpE* | ABC superfamily (membrane),sn-glycerol 3-phosphate transport protein |
| *STM0857* |  | putative acyl-CoA dehydrogenase |
| *STM3537* | *glgX* | glycosyl hydrolase |
| *STM1691* | *pspF* | transcription activator |
| *STM3377* |  | putative nitrate reductase |
| *STM2161* |  | putative inner membrane protein |
| *STM3390* | *acrE* | transmembrane protein affecting septum formation and cell membrane permeability |
| *STM3664* | *malS* | alpha-amylase |
| *STM4486* | *yjgB* | putative alcohol dehydrogenase |
| *STM4080* |  | putative ribulose-5-phosphate 3-epimerase |
| *STM3086* | *speA* | arginine decarboxylase |
| *STM1376* | *lppB* | putative methyl-accepting chemotaxis protein |
| *STM0526* | *ylbA* | putative glyoxylate utilization |
| *STM2766* |  | putative cytoplasmic protein |
| *STM4272* |  | putative inner membrane protein |
| *STM4528* |  | putative inner membrane protein |
| *STM3879* | *yieN* | paral putative regulator protein |
| *STM4045* | *rhaD* | rhamnulose-1-phosphate aldolase |
| *STM2669* | *tyrA* | bifunctional: chorismate mutase T; prephenate dehydrogenase |
| *STM1010* |  | Gifsy-2 prophage |
| *STM3276* | *yhbW* | putative alkanal monooxygenase |
| *STM3427.S* | *rpsN* | 30S ribosomal subunit protein S14 |
| *STM3810* | *yidQ* | putative outer membrane lipoprotein |
| *STM0091* | *pdxA* | NAD-dependent dehydrogenase/carboxylase |
| *PSLT069* | *psiB* | Plasmid SOS inhibition |
| *STM4411* | *ytfP* | putative cytoplasmic protein |
| *STM4375* | *yjfM* | putative inner membrane protein |
| *STM3093* | *endA* | DNA-specific endonuclease I |
| *STM3075* |  | putative ABC-type cobalt transport system, ATPase component |
| *STM1977* | *fliN* | flagellar biosynthesis, component of motor switch and energizing |
| *STM3261* |  | galactitol-1-phosphate dehydrogenase |
| *STM3058* | *pepP* | proline aminopeptidase P II |
| *STM3109* | *yggH* | putative S-adenosylmethionine-dependent methyltransferase |
| *STM3647* | *yiaF* | putative outer membrane lipoprotein |
| *STM1793* |  | putative cytochrome oxidase, subunit II |
| *STM1443* | *ydhI* | putative inner membrane protein |
| *STM3189* | *ygiD* | putative cytoplasmic protein |
| *STM3628* | *dppC* | ABC superfamily (membrane), dipeptide transport protein 2 |
| *STM1626* | *trg* | methyl-accepting chemotaxis protein III, ribose and galactose sensor receptor |
| *STM4524* | *hsdS* | specificity determinant for hsdM and hsdR |
| *STM1932* | *ftnB* | ferritin-like protein |
| *STM0876* | *ybjN* | putative cytoplasmic protein |
| *STM1986* | *yedP* | putative hydrolase of the HAD superfamily |
| *STM0935* | *poxB* | pyruvate dehydrogenase/oxidase FAD and thiamine PPi cofactors |
| *STM3690* |  | putative inner membrane lipoprotein |
| *STM1985* | *dsrA* | a small RNA antisilencer of the H-HS-silenced rdsA gene in E. coli |
| *PSLT063* |  | putative cytoplasmic protein |
| *STM3293* | *secG* | preprotein translocase IISP family, auxillary membrane component |
| *STM3977* | *rfaH* | transcriptional activator affecting biosynthesis of lipopolysaccharide core, F pilin, and haemolysin |
| *STM1187* | *rluC* | 23S rRNA pseudouridylate synthase |
| *STM4594* | *sthA* | putative fimbrial chaparone protein |
| *STM2113* | *wcaC* | putative glycosyl transferase in colanic acid biosynthesis |
| *STM0777* |  | putative inner membrane protein |
| *STM0692* |  | putative transcriptional regulator, LysR family |
| *PSLT007* |  | putative outer membrane protein |
| *STM3821* | *torD* | cytoplasmic chaperone which interacts with TorA |
| *STM1870* |  | Homology to recE (exoVIII) in E. coli |
| *STM0765* |  | putative cation transporter |
| *STM1184* | *flgL* | flagellar biosynthesis; hook-filament junction protein |
| *STM4362* | *hflX* | putative GTP-ase, together with HflCK possibly involved in phage lambda cII repressor stability |
| *PSLT029* |  | putative cytoplasmic protein |
| *STM4287.S* | *phnO* | putative regulatory protein |
| *STM3833* |  | putative mandelate racemase / muconate lactonizing enzyme family |
| *STM3673* | *yiaO* | putative dicarboxylate-binding periplasmic protein |
| *STM4479* | *yjgP* | putative permease |
| *STM4297* | *melR* | regulator of melibiose operon (AraC/XylS family) |
| *STM0495* | *ybaK* | putative cytoplasmic protein |
| *STM0096* | *hepA* | RNA polymerase associated protein, putative SNF2 family RNA helicase |
| *STM3415* | *rpoA* | RNA polymerase, alpha subunit |
| *STM4151* | *rplJ* | 50S ribosomal subunit protein L10 |
| *STM1016* |  | Gifsy-2 prophage |
| *STM1253* |  | putative inner membrane protein |
| *STM0565* |  | putative periplasmic protein |
| *STM0636* | *ybeD* | putative cytoplasmic protein |
| *STM1931* | *araH* | putative intracellular protease/amidase |
| *STM0257* |  | putative drug efflux protein (perhaps for chloramphenicol) |
| *STM0195* | *stfA* | putative fimbrial subunit |
| *STM3001* | *thyA* | thymidylate synthetase |
| *STM0035* |  | putative arylsulfatase |
| *STM3156* |  | putative cytoplasmic protein |
| *STM2177* |  | putative flutathione S-transferase |
| *STM0369* | *prpC* | putative citrate synthase |
| *STM1567* | *adhP* | alcohol dehydrogenase, propanol preferring |
| *STM3712* | *rfaC* | heptosyl transferase I |
| *STM4457* |  | putative transposase |
| *STM0735* | *sdhB* | succinate dehydrogenase, Fe-S protein |
| *STM0773* | *galM* | galactose-1-epimerase (mutarotase) |
| *STM0731* |  | putative inner membrane protein |
| *STM1105* | *hpaH* | 4-hydroxyphenylacetate catabolism |
| *STM1406* | *ssaG* | Secretion system apparatus |
| *STM0964* | *dmsA* | anaerobic dimethyl sulfoxide reductase, subunit A |
| *STM2463* | *eutE* | putative aldehyde oxidoreductase in ethanolamine utilization |
| *STM2079* | *wzzB* | regulator of length of O-antigen component of lipopolysaccharide chains |
| *STM3219* | *fadH* | 2,4-dieonyl-coa reductase |
| *STM2176* |  | putative glutathione S-transferase |
